# The “Genomic Storm” Induced by Bacterial Endotoxin Is Calmed by a Nuclear Transport Modifier That Attenuates Localized and Systemic Inflammation

**DOI:** 10.1371/journal.pone.0110183

**Published:** 2014-10-20

**Authors:** Antonio DiGiandomenico, Ruth Ann Veach, Jozef Zienkiewicz, Daniel J. Moore, Lukasz S. Wylezinski, Martha A. Hutchens, Jacek Hawiger

**Affiliations:** 1 Department of Microbiology and Immunology Vanderbilt University School of Medicine, Nashville, Tennessee, United States of America; 2 Immunotherapy Program at Vanderbilt University School of Medicine, Nashville, Tennessee, United States of America; 3 Department of Medicine, Division of Allergy, Pulmonary and Critical Care Medicine, Vanderbilt University School of Medicine, Nashville, Tennessee, United States of America; 4 Department of Pediatrics, Ian Burr Division of Endocrinology and Diabetes, Vanderbilt University School of Medicine, Nashville, Tennessee, United States of America; 5 Department of Pathology, Microbiology and Immunology, Vanderbilt University School of Medicine, Nashville, Tennessee, United States of America; 6 Department of Molecular Physiology and Biophysics, Vanderbilt University School of Medicine, Nashville, Tennessee, United States of America; Virginia Polytechnic Institute and State University, United States of America

## Abstract

Lipopolysaccharide (LPS) is a potent microbial virulence factor that can trigger production of proinflammatory mediators involved in the pathogenesis of localized and systemic inflammation. Importantly, the role of nuclear transport of stress responsive transcription factors in this LPS-generated “genomic storm” remains largely undefined. We developed a new nuclear transport modifier (NTM) peptide, cell-penetrating cSN50.1, which targets nuclear transport shuttles importin α5 and importin β1, to analyze its effect in LPS-induced localized (acute lung injury) and systemic (lethal endotoxic shock) murine inflammation models. We analyzed a human genome database to match 46 genes that encode cytokines, chemokines and their receptors with transcription factors whose nuclear transport is known to be modulated by NTM. We then tested the effect of cSN50.1 peptide on proinflammatory gene expression in murine bone marrow-derived macrophages stimulated with LPS. This NTM suppressed a proinflammatory transcriptome of 37 out of 84 genes analyzed, without altering expression of housekeeping genes or being cytotoxic. Consistent with gene expression analysis in primary macrophages, plasma levels of 23 out of 26 LPS-induced proinflammatory cytokines, chemokines, and growth factors were significantly attenuated in a murine model of LPS-induced systemic inflammation (lethal endotoxic shock) while the anti-inflammatory cytokine, interleukin 10, was enhanced. This anti-inflammatory reprogramming of the endotoxin-induced genomic response was accompanied by complete protection against lethal endotoxic shock with prophylactic NTM treatment, and 75% protection when NTM was first administered after LPS exposure. In a murine model of localized lung inflammation caused by direct airway exposure to LPS, expression of cytokines and chemokines in the bronchoalveolar space was suppressed with a concomitant reduction of neutrophil trafficking. Thus, calming the LPS-triggered “genomic storm” by modulating nuclear transport with cSN50.1 peptide attenuates the systemic inflammatory response associated with lethal shock as well as localized lung inflammation.

## Introduction

Bacterial endotoxin, known as lipopolysaccharide (LPS), is one of the most potent microbial virulence factors in the pathogenesis of localized and systemic inflammation caused by Gram-negative bacteria [1]. LPS is the primary component of the Gram-negative bacterial outer membrane and the most proinflammatory of all bacterial pathogen-associated molecular patterns recognized by Toll-like receptors (TLRs). TLRs are expressed on multiple cell types, including myeloid and lymphoid cells, vascular endothelial cells, and respiratory epithelial cells [2], [3]. Binding of LPS to its cognate receptor, TLR4, induces robust signaling to the nucleus mediated by a cascade of signal transducers engaged in a stream of protein-protein interactions and posttranslational modifications [4], culminating in nuclear translocation of NF-κB along with other stress-responsive transcription factors (SRTFs), including activator protein-1 (AP-1), nuclear factor of activated T cells (NFAT), and signal transducer and activator of transcription 1 (STAT-1) [5]. These SRTFs, either alone or in various combinations, regulate the genomic response to Gram-negative bacteria and other microbial agents [5]. Similarly, SRTFs respond to signaling pathways emanating from cytokine/chemokine receptors [6], [7].

SRTFs and other nuclear proteins larger than 45 kDa are transported to the nucleus by a set of adaptor proteins known as importins (Imp)/karyopherins α which in tandem with importin β1, ferry the SRTF cargo to the nucleus [5], [6], [8], [9]. Therein, they activate a plethora of genes that encode inflammatory cytokines and chemokines, signal transducers (cyclooxygenase, nitric oxide synthase), and cell adhesion molecules, a response denoted as a “genomic storm”. The concept of a “genomic storm” induced by trauma and burns in critically injured patients was extended to subjects challenged with bacterial endotoxin, and therefore represents a fundamental human response to severe inflammatory stress [10]. A tidal wave of gene expression raises blood levels of cytokines and chemokines and mobilizes expression of other mediators. Cumulatively, these products of genomic reprogramming induce fever, endothelial instability and detachment, disseminated intravascular coagulation, acute lung inflammation (ALI), acute respiratory distress syndrome (ARDS), and multiple organ dysfunction, culminating in vascular collapse refractory to fluid resuscitation (septic shock), and death [11], [12].

Though prompt initiation of anti-microbial therapy is crucial in limiting the extent of Gram-negative bacterial infections [13], residual circulating LPS can sustain production of inflammatory mediators by blood leukocytes and microvascular endothelial cells [14]. Given the plethora of proinflammatory mediators that are produced [15], focusing therapy on single inflammatory molecules will likely not alleviate the morbidity associated with this disease [16]. Rather, a more comprehensive countermeasure to reduce the flow of SRTFs to the nucleus would be preferable. Therefore, we hypothesized that targeting the nuclear transport machinery, which integrates multiple signaling pathways emanating from endotoxin-stimulated TLR4 and from subsequently produced cytokines and chemokines [5], would calm the “genomic storm”. Thus, it would be plausible to reduce lethal endotoxic shock (systemic inflammation) while also attenuating expression of inflammatory mediators in the lungs (localized inflammation). Using computer-based analysis of a public database, we identified the regulatory elements in 46 human genes that encode mediators of inflammation. These regulatory elements are recognized by SRTFs dependent on nuclear translocation mediated by importins α and β [5], [7]. To test our hypothesis, we used the next generation NTM, cSN50.1, a highly soluble 28 amino acid cell-penetrating peptide [6] that targets Imp α5 and Imp β1 [9]. We assessed its impact on the inflammatory transcriptome of primary bone marrow-derived macrophages stimulated with LPS. Then, we compared the action of NTM in primary macrophages with *in vivo* analysis in murine models of systemic and localized inflammation induced by LPS.

Here we report that the modulating nuclear transport with the cell-penetrating NTM, cSN50.1 peptide, leads to selective attenuation of the LPS-induced transcriptome of murine bone marrow-derived macrophages and striking suppression of LPS-induced systemic endotoxic shock and localized lung inflammation. These results support the concept of targeting nuclear import of transcription factors as a means to control the LPS-induced “genomic storm” and its resultant inflammatory responses.

## Materials and Methods

### Peptide synthesis and purification

Highly soluble cell-penetrating NTM peptide cSN50.1 (Table 1), was synthesized, purified, and filter-sterilized as described elsewhere [6], [9].

**Table 1:** Amino acid sequence of cSN50.1 peptide and its congeners SN50 and cSN50.

Peptide	Sequence	MW [Da]	Solubility in Water	
			[mg/mL]	[mM]
SN50	**AAVALLPAVLLALLAP** *VQRKRQKLMP*	2781	13	4.7
cSN50	**AAVALLPAVLLALLAP**CY*VQRKRQKLMP*C	3149	38	12.1
cSN50.1	**AAVALLPAVLLALLAP**C*VQRKRQKLMP*C	2986	100	33.5

Fragment-linked peptides comprising the Signal Sequence Hydrophobic Region of Fibroblast Growth Factor 4 (bolded) and the NLS region of NFκB1/p50 (italicized) were analyzed for their solubility in water. In cSN50 and cSN50.1, an intra-molecular disulfide bond is formed between the two cysteines, which cyclizes the NLS motif.

### Transcription Factor/Targeted Gene pair selection

Human genes encoding the cytokines, chemokines, receptors and growth factors studied in this manuscript, were analyzed for specific binding sites of transcription factors whose nuclear translocation was previously shown to be modulated by NTM (Table 2). The prediction process was conducted based on the presence of a binding site in the promoter region of the targeted gene. To accomplish this task we employed the UCSC Genome Browser publicly available on the website of the Center for Biomolecular Science and Engineering at the University of California Santa Cruz (UCSC Genome Bioinformatics, http://genome.ucsc.edu).

**Table 2:** Genes encoding mediators of inflammation are regulated by transcription factors that require nuclear transport shuttles targeted by NTM.

	Transcription Factor(MW/kDa)	Nuclear TargetingMotif	# of targetedgenes	Cytokines and GrowthFactors																				Chemokines																	Receptors/Transporters								
				Il1a	Il1b	Il6	Il9	Il10	Il12p40	Il12p70	Il13	Il15	Il17	Il18/IGIF	Ifng	Lif	Lta	Tnf	Spp1	G-csf/Csf3	M-csf/Csf1	Gm-csf/Csf2	Vegf	Ccl2/MCP-1	Ccl3/MIP-1α	Ccl4/MIP-1β	Ccl5/RANTES	Ccl7/MCP-3	Ccl8/MCP-2	Ccl17/TARC	Ccl19/MIP-3β	Ccl20/MIP-3α	Ccl22/MDC	Ccl24/MPIF-2	Cx3cl1/ABCD-3	Cxcl2/MIP-2α	Cxcl5/LIX	Cxcl9/MIG	Cxcl10/IP-10	Cxcl11/IP-9	Cxcr5/CD185	Ccr2/CD192	Ccr3/CD193	Ccr7/CD197	Ccr10/GPR2	Il6ra	Il6st	Tnfrsf1b	Abcf1
1	NF-κB1/p50 (48)	mNLS	46	+	+	+	+	+	+	+	+	+	+	+	+	+	+	+	+	+	+	+	+	+	+	+	+	+	+	+	+	+	+	+	+	+	+	+	+	+	+	+	+	+	+	+	+	+	+
2	STAT-1 (87)	uNLS	34	+			+	+	+	+	+		+	+	+	+	+	+	+	+	+	+	+	+	+	+		+	+								+		+	+	+	+	+	+	+	+	+	+	+
3	AP-1 c-Jun (36)	bNLS	32	+	+	+	+				+		+	+		+	+	+	+	+	+	+	+	+	+	+	+	+					+		+				+	+	+	+	+	+	+	+		+	+
4	Rel A (60)	mNLS	32	+	+	+	+	+	+		+				+	+	+	+		+	+	+		+			+	+	+	+	+	+		+	+	+	+	+	+	+	+						+	+	+
5	NF-κB2/p52 (49)	mNLS	31	+	+	+		+	+	+					+	+	+	+			+	+		+	+		+	+	+	+		+	+	+	+	+	+	+	+		+					+	+	+	+
6	c-Rel (68)	mNLS	26		+	+	+	+			+	+				+	+	+		+	+			+			+	+		+		+		+		+	+	+	+	+	+			+			+	+	
7	nSREBP1 (51)	bHLH	19										+	+	+	+	+	+		+	+	+	+						+		+				+			+	+		+	+			+				+
8	NFATc4 (96)	mNLS	15		+	+	+	+			+		+			+	+	+		+	+								+					+											+				+
9	NFATc1 (77)	mNLS	14		+	+	+	+			+					+	+	+		+	+								+					+											+				+
10	NFATc2 (100)	mNLS	14		+	+	+	+			+					+	+	+		+	+								+					+											+				+
11	NFATc3 (116)	mNLS	14		+	+	+	+			+					+	+	+		+	+								+					+											+				+
12	NRF-2 (68)	mNLS	6					+								+	+	+																							+								+

Analysis was performed *in silico* using The UCSC Genome Browser (The UCSC Genome Bioinformatics). mNLS - classical monopartite Nuclear Localization Sequence; bNLS - nonclassical bipartite Nuclear Localization Sequence; uNLS - unconventional, structural or dimer-specific Nuclear Localization Sequence; bHLH - basic helix-loop-helix motif; “+” - the transcription factor specific binding site is present in the gene promoter.

### Isolation and cultivation of bone marrow-derived macrophages (BMDMs)

Bone marrow cells were isolated from femurs and tibias of C57BL/6 mice and suspended in Dulbecco's Modified Eagle Medium supplemented with 10% FBS, 10 mM HEPES, 100 U/ml penicillin, 100 µg/ml streptomycin, and 20% L929-conditioned medium. Non-adherent cells were removed and culture media replaced every three days. Cells were used in experiments after 10 days of culture for up to 2 weeks after maturation. Prior to use in experiments, culture purity of adherent cells was verified by fluorescence-activated cell sorting where ≥95% were MAC3^+^, CD11b^+^, CD3^–^, CD11c^–^ and B220^–^. Viability was ≥80% as determined by trypan blue exclusion.

### LPS treatment of BMDMs and quantitative real-time PCR (qRT-PCR)

BMDMs were left unstimulated for preparation of control RNA, or stimulated with 2 ng/ml LPS from *E. coli* O127:B8 (Sigma) and concurrently treated with cSN50.1 (30 µM) or saline (diluent). After incubation at 37°C for 6 h, RNA was prepared from cells using the Qiagen RNeasy Kit (Qiagen) and converted to cDNA using the RT^2^ First Strand Kit, then analyzed using the RT^2^ Profiler PCR array system (Qiagen) according the manufacturer’s instructions.

### Ethics statement

All animal handling and experimental procedures were performed in strict accordance with the recommendations in the Guide for the Care and Use of Laboratory Animals of the National Institutes of Health. The protocol was approved by the Vanderbilt University Animal Care and Use Program (Permit Number: A3227-01), which has been accredited by the American Association of Accreditation of Laboratory Animal Care International (file #000020). Animals were housed in groups of five in the animal care facility of Vanderbilt University in a 12 hour light/dark cycle. Regular rodent chow and water were provided *ad libitum*. After administration of inflammatory agonists, mice are carefully monitored and any that exhibit end−stage symptoms consistent with acute toxic shock are euthanized as soon as it is apparent they will not recover.

### Murine models of LPS-induced systemic (lethal shock) and localized (lung) inflammation

Randomized groups of five female C57BL/6 mice (The Jackson Laboratory) 8–12 weeks of age (∼20 g weight) and LPS from *E. coli* O127:B8 (Sigma) were used in all animal experiments. To evaluate the protective efficacy of NTM (cSN50.1 peptide) against systemic inflammation, two models of lethal endotoxic shock were used: high-dose LPS; or low-dose LPS under conditions of metabolic stress imposed by 2-amino-2-deoxy-D-galactosamine (D-Gal), which sensitizes mice to the proinflammatory action of LPS [17]. In the high-dose LPS model, 800 µg LPS in 0.2 ml saline was administered by intraperitoneal (i.p.) injection. In the LPS+D-Gal model, mice were injected i.p. with 1 µg of LPS and 20 mg of D-Gal, each in 0.2 ml saline. Two NTM treatment protocols, prophylactic and therapeutic, were tested in the high-dose LPS model. In the prophylactic protocol, mice were given NTM (cSN50.1 peptide, 0.66 mg/injection), or diluent (saline) by i.p. injections of 0.2 ml at 30 min before and 30, 90, 150, 210, 360 and 720 min after LPS challenge, while in the therapeutic protocol, NTM was administered at 15, 90, 150, 210, 360, and 720 min post-LPS challenge. Blood samples (∼40 µL) were collected from the saphenous vein in heparinized tubes (Sarstedt) before and at 2, 4, 6 and 24 h post-LPS challenge. All injected reagents were sterile and prepared in pyrogen-free saline. These experiments are based on the death of animals as an experimental endpoint, so mice were allowed to progress to a moribund state before being euthanized by isoflurane asphyxiation. Since multiple organ systems are affected in the mechanism of systemic inflammation, any pain medication may inadvertently interfere with the progression of endotoxic shock. Therefore, we could not use agents that alleviate pain. However, we attempted to minimize the amount of pain experienced by the animals by closely monitoring mice, at least hourly for the first 24 hours and three times a day thereafter, and euthanizing any mice that exhibit end-stage symptoms consistent with acute toxic shock (lack of reaction to cage motion, or any of the following signs: ataxia, paralysis, cyanosis, or severe respiratory distress) as soon as it is apparent they will not recover. Any surviving mice were euthanized after 72 h.

For induction of localized acute lung inflammation, intranasal instillations of 50 ng of LPS in 50 µL saline (25 µL/nostril) were performed under ketamine/xylazine anesthesia (0.2 ml of 6.7 mg/ml ketamine and 1.3 mg/ml xylazine administered by i.p. injection). Mice were treated with NTM (cSN50.1 peptide, 0.66 mg/injection) or diluent (saline) administered by i.p. injections of 0.2 ml at 30 min before and at 30, 90, and 120 min after LPS challenge. Six hours post-LPS challenge, mice were euthanized by isoflurane asphyxiation. Bronchoalveolar lavage (BAL) collection and differential cell counts were performed as previously described [18]. For comparison, BAL was also collected from naïve mice not exposed to any intranasal instillation before euthanasia.

### Analysis of chemokines, cytokines, and growth factors in BAL fluid and plasma

The effect of cSN50.1 on expression of chemokines, cytokines and growth factors was analyzed in cell-free BAL fluid or blood plasma from mice by cytometric bead array (BD BioSciences) or a 32-analyte Milliplex mouse panel (Millipore) according to the manufacturers’ instructions. Analytes included eotaxin, granulocyte colony-stimulating factor (G-CSF), granulocyte macrophage colony-stimulating factor (GM-CSF), interferon gamma (IFN-γ), interleukin (IL) −1α, IL-1β, IL-2, IL-3, IL-4, IL-5, IL-6, IL-7, IL-9, IL-10, IL-12 (p40), IL-12 (p70), IL-13, IL-15, IL-17, IFN-γ-induced protein 10 (IP-10), keratinocyte chemoattractant (KC), leukemia inhibitory factor (LIF), LPS-induced CXC chemokine (LIX), macrophage colony-stimulating factor (M-CSF), monocyte chemoattractant protein-1 (MCP-1), monokine induced by gamma interferon (MIG), macrophage inflammatory protein (MIP) −1α, MIP-1β, MIP-2, regulated upon activation normal T-cell expressed, and presumably secreted (RANTES), tumor necrosis factor alpha (TNF-α), and vascular endothelial growth factor (VEGF).

### Statistical analyses

Threshold cycle values from qRT-PCR were exported to Excel and analyzed using the Qiagen web-based PCR Array Data Analysis Software. Genes showing a >0.5 log fold change versus control were considered significant. Other data analysis and statistical calculations were performed using Prism (GraphPad). Cell counts and chemokine, cytokine, and growth factor levels in BAL were compared using the non-parametric Mann–Whitney *U* test. Survival data were analyzed by the log-rank test. Cytokine, chemokine and growth factor levels in plasma collected from the same animals at different time points were evaluated by repeated measures two-way analysis of variance with Sidak’s post-test. A *p* value of <0.05 was considered significant.

## Results

### Design and characterization of the next generation NTM, cSN50.1 peptide, and its previous congeners

A fragment-linked peptide strategy to analyze signal-dependent nuclear transport is based on fusing a motif from the nuclear localization sequence (NLS) region of the NF-κB1/p50 subunit with the signal sequence hydrophobic region (SSHR) of human fibroblast growth factor 4 [7], [19]. NLS- and SSHR-containing NTMs (SN50, cSN50 and cSN50.1; see Table 1 for their composition and solubility) were designed to function at the nuclear transport level by binding to importins α during stimulus-initiated signaling and thereby modulate nuclear import of NLS-bearing SRTFs. The SSHR motif allows peptides to cross the plasma membrane of cells in culture or in experimental animals through an ATP- and endosome-independent mechanism [20]. In this study, we used cSN50.1, an improved version of our previously described cell-penetrating NTM peptides [6], [9], [21], [22] that shows increased solubility (100 mg/ml) in water, compared to cSN50 (38 mg/ml) and SN50 (13 mg/ml) (see Table 1), thereby increasing its potency [6], [21]. The functional utility of NTMs has been reported in numerous preclinical models of inflammation caused by microbial and autoimmune insults [18], [21]–[26]. Most recently, and unexpectedly, we found that cSN50.1 not only modulates nuclear transport of SRTFs such as NF-κB, but also sterol regulatory element-binding protein (SREBP) transcription factors that regulate lipid homeostasis [6].

### NTM has a calming effect on the “genomic storm” induced by LPS in primary macrophages

Recently, it was shown that injection of bacterial endotoxin into healthy adult volunteers was associated with a robust genomic response in combined blood leukocyte populations [10]. Among the genes reported in that study, several encoded cytokines, chemokines, and their receptors as reflected by transcriptome analysis. We used a publicly available database to match genes encoding mediators of inflammation with transcription factors containing nuclear targeting motifs known to require nuclear transport shuttles recognized by cSN50.1 [6] (Table 2). We found that all 46 proinflammatory genes are potentially regulated by NF-κB1, 34 genes by STAT-1, 32 genes by c-Jun, and 19 genes by SREBP1. Most of these genes are combinatorially regulated by two or more transcription factors that are transported to the nucleus as monomers or homodimers/heterodimers by importins α and β. Their transport function is modulated by NTMs that encompass cell penetrating SN50, cSN50, and cSN50.1 peptides [6], [9] (Table 1). It is thus plausible that cSN50.1 peptide can suppress expression of multiple proinflammatory genes by modulating nuclear transport of transcription factors analyzed in Table 2.

**Figure 1:**
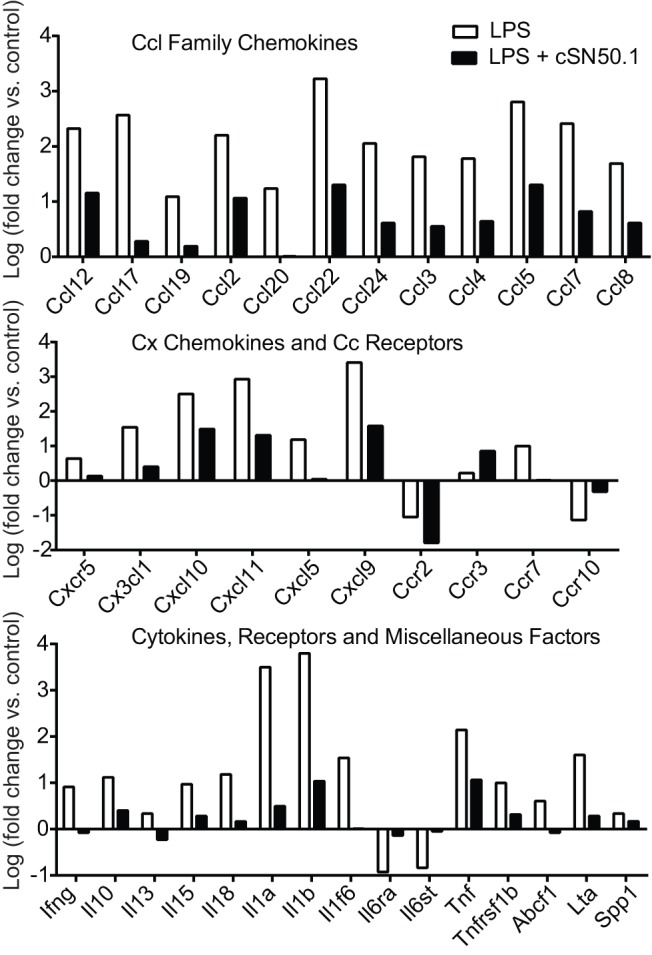
qRT-PCR-based gene expression analysis of LPS-challenged BMDMs in the absence or presence of NTM. *Ccl* family chemokines, *Cx* chemokines and *Cc* receptors, and cytokines, receptors and miscellaneous factors from an 84 gene array exhibiting a >0.5 log fold change in LPS-stimulated primary macrophages compared to unstimulated control cells.

We tested this hypothesis through analysis of the proinflammatory transcriptome in bone marrow-derived macrophages. These primary cells are one of the main myeloid lineage targets of LPS [27]. A mouse inflammatory cytokine/chemokine and receptor PCR array allowed us to assess the effects of NTM on 84 genes compared to untreated controls. Remarkably, NTM modified expression of 37 of the 84 genes tested. While NTM suppressed gene expression in LPS-activated inflammatory pathways (Figure 1), it did not alter gene expression of five housekeeping genes (*Gusb*, *Hprt1*, *Hsp90ab1*, *Gapdh*, A*ctb*, <0.5 fold change versus control, not shown). Genes encoding most cytokines and CC chemokines were down-regulated by NTM whereas those for most CC chemokine receptors, cytokine receptors, and CX chemokines and their receptors were not affected. There were a few notable exceptions: LIX/CXCL5, MIG/CXCL9, and IP-10/CXCL10. Importantly, this suppression of genomic changes by NTM in primary macrophages challenged with LPS was not associated with changes in cell viability (data not shown), indicating that NTM has no adverse effect on cell growth. Thus, targeting nuclear transport pathways for LPS-activated SRTFs with cSN50.1 peptide prevented a “genomic storm” by reducing transcription of a wide array of genes that encode mediators of inflammation in primary macrophages.

### NTM attenuates LPS-induced systemic inflammation in the form of lethal endotoxic shock accompanied by a burst of proinflammatory cytokines and chemokines in blood

**Figure 2:**
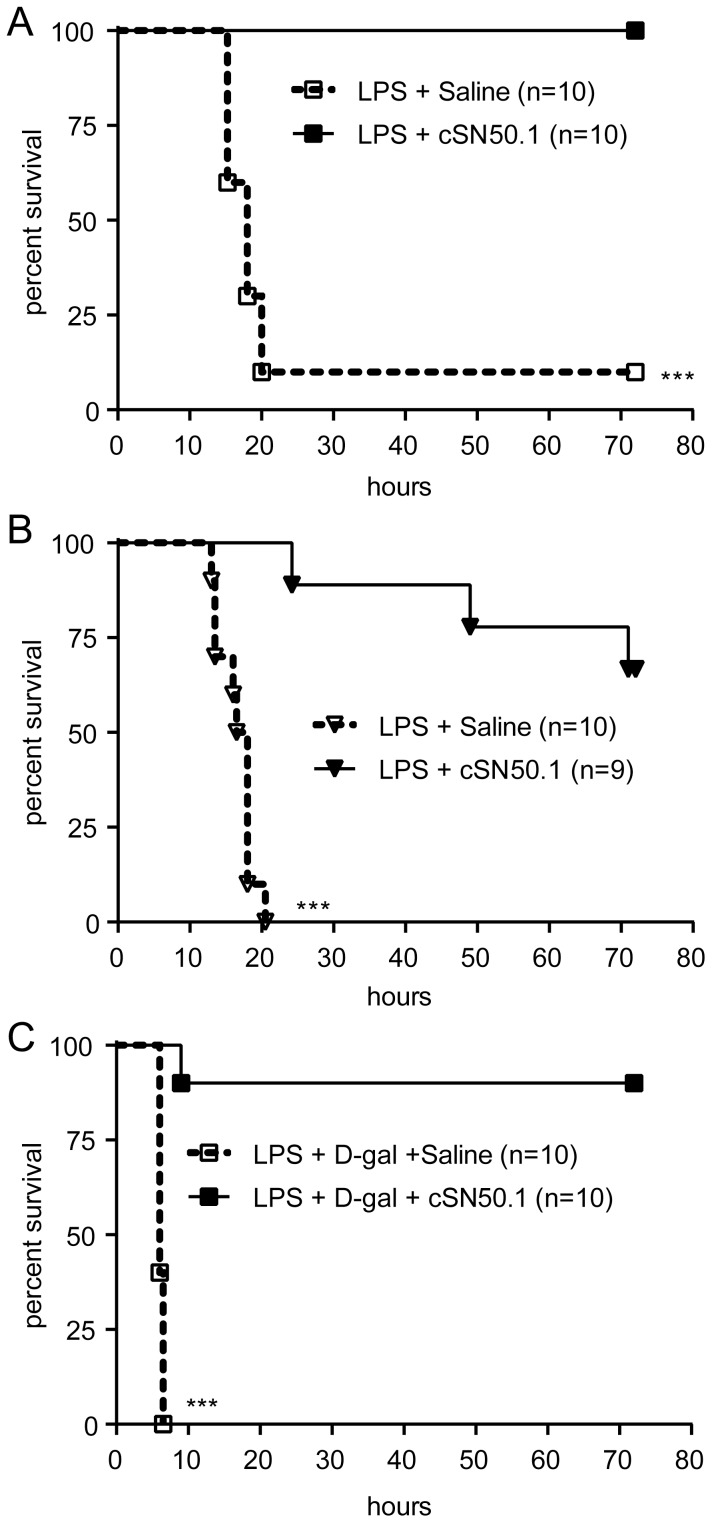
NTM treatment enhances survival from lethal endotoxic shock. Survival curves for mice challenged i.p. with high-dose LPS (A and B) or low-dose LPS+D-Gal (C). In (A) and (C) mice were administered the first NTM (cSN50.1 peptide) treatment 30 min before LPS challenge (prophylactic protocol), while in (B), the first treatment was administered 15 min after LPS challenge (therapeutic protocol). Saline injections were administered to control mice challenged with LPS following the same treatment schedule in each protocol, as described in “Materials and Methods”. ***p<0.0005 by log rank test.

After analysis of the primary macrophage response to LPS modulated by NTM, we studied its effect on two models of LPS-induced systemic inflammation exemplified by lethal shock. In a high-dose LPS model, both prophylactic and therapeutic NTM treatment protocols were employed. In the prophylactic protocol, the first dose of NTM was administered before LPS challenge while in the therapeutic protocol, NTM treatment was begun after LPS administration. As shown in Figure 2A, animals treated with NTM by the prophylactic protocol in the high-dose LPS model were completely protected, compared to only 10% survival in saline-treated control animals (*p*<0.0001). Strikingly, when a therapeutic NTM treatment protocol was employed in the high-dose LPS model, 75% of mice survived, compared to 100% mortality in the saline-treated control group (Figure 2B, *p*<0.0001). Thus, NTM was highly effective against high-dose LPS with both prophylactic and therapeutic protocols.

To further evaluate the effectiveness of the NTM, we tested its anti-inflammatory effect under conditions of metabolic stress imposed by D-Gal, which sensitizes mice to LPS [17]. In contrast to the high-dose LPS model, which requires at least 800 µg of LPS per 20 g mouse for induction of lethal shock, only 1 µg of LPS per 20 g mouse is required for lethality when mice are metabolically stressed with D-Gal, and death occurs much more rapidly (5–7 h after low-dose LPS+D-gal compared to 16–20 h post-LPS in the high-dose model). Consistent with results from the high-dose LPS shock model, prophylactic NTM treatment afforded robust protection (90%) in mice challenged with LPS+D-Gal. In contrast, no mice survived in the control group treated with saline (Figure 2C, *p*<0.0005).

**Figure 3:**
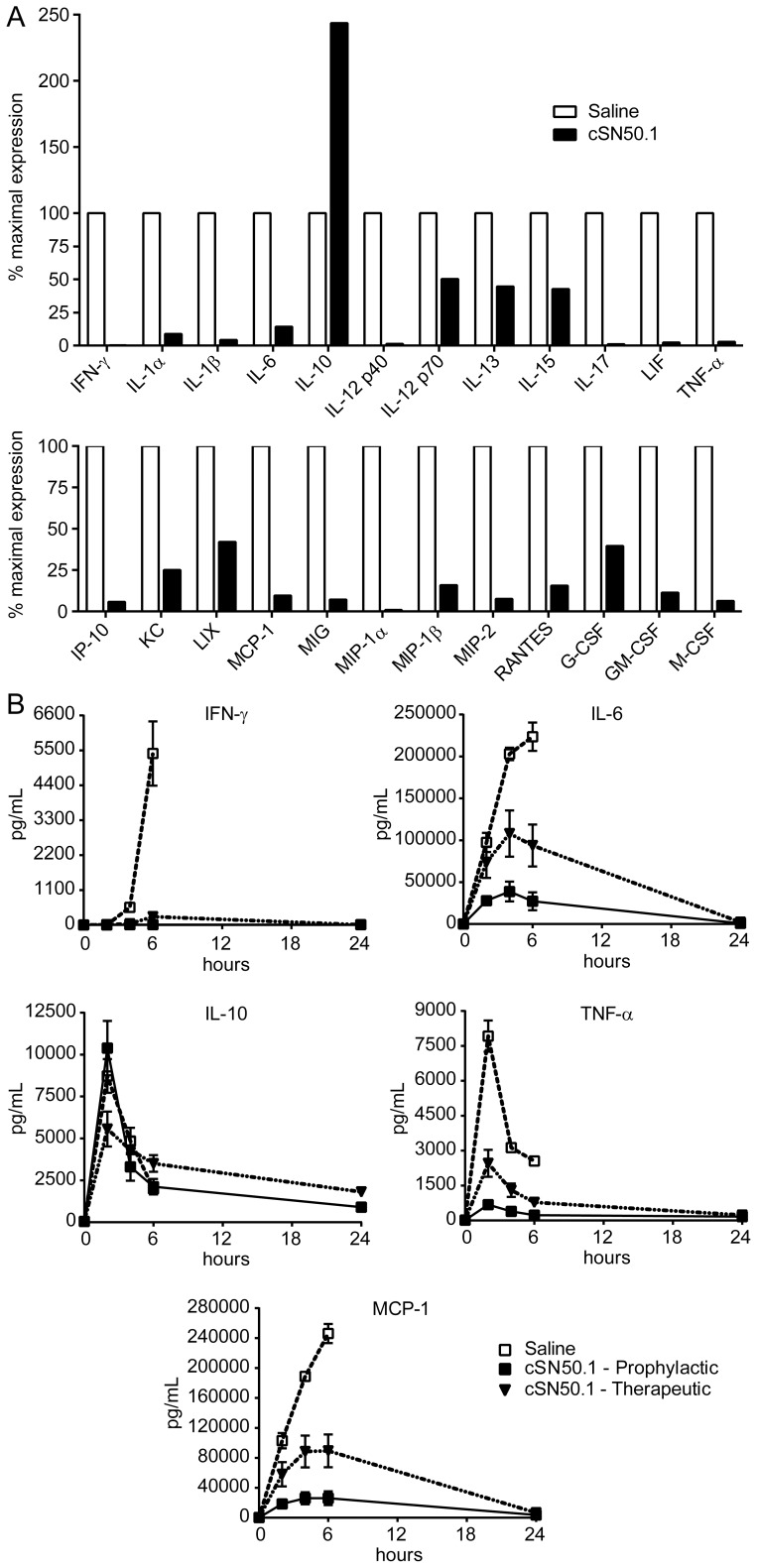
NTM treatment reduces plasma levels of multiple cytokines, chemokines and growth factors induced by LPS. (A) Wild type C57BL/6 mice were challenged i.p. with a lethal dose of LPS (800 µg) and treated with i.p. injections of NTM (cSN50.1 peptide) or diluent (saline) following a prophylactic protocol as in Figure 4A. Blood was collected at baseline and 2 or 6 h after LPS challenge and a multiplex assay was used to measure 32 analytes in plasma. Twenty-four analytes were significantly altered by NTM treatment, as determined by repeated measures two-way analysis of variance with Sidak’s post-test. Twenty-three were reduced, while anti-inflammatory IL-10 was increased by NTM treatment. Results are shown as the % inhibition or increase by NTM compared to saline control set to 100% at the time point demonstrating maximal expression for that analyte. *n* = 10 animals/group. (B) Comparison of prophylactic and therapeutic NTM treatment protocols on selected plasma cytokine and chemokine levels in the high-dose LPS model of endotoxic shock. Data are presented as mean ± standard error, *n* = 5 −10 animals/group.

We analyzed the striking gain in survival of NTM-treated mice that were challenged with LPS in the context of systemic proinflammatory cytokine and chemokine production. As expected, in the prophylactic protocol, NTM treatment engendered significant inhibition of 11 out of 13 proinflammatory cytokines whose plasma levels were increased by LPS challenge (Figure 3A, upper). An exception was the anti-inflammatory cytokine, IL-10, which was elevated >2 fold in NTM-treated animals. In parallel, a wide array of LPS-elevated chemokines and growth factors was suppressed in NTM-treated mice (Figure 3A, lower). Overall, of 26 cytokines, chemokines and growth factors elevated in plasma after LPS challenge, 23 were reduced by NTM treatment, two were unchanged (eotaxin/CCL11 and IL-5, not shown) and one was increased (IL-10). Plasma levels of the remaining six of the 32 analytes tested were not increased by administration of LPS (not shown). These results are consistent with the genomic response of primary macrophages in the qRT-PCR analysis (see Figure 1), indicating that NTM, under the conditions of these *in vivo* experiments, suppresses expression of multiple mediators of inflammation. A comparable trend in suppression of proinflammatory cytokines and chemokines TNF-α, IL-6, IFN-γ and MCP-1 was observed in mice treated with NTM when employing the therapeutic protocol, albeit to a lesser degree. Interestingly in the therapeutic protocol, IL-10 is suppressed instead of enhanced by NTM (Figure 3B), consistent with suppression of the *Il10* gene in primary macrophages (see Figure 1).

Cumulatively, our results indicate that NTM affords robust protection of mice from LPS-induced systemic inflammation in two distinct models of lethal shock. Concurrently, we noted a striking reprogramming of the inflammatory response that entails suppression of many proinflammatory cytokines and chemokines in plasma.

### NTM attenuates proinflammatory cytokine and chemokine expression after direct airway exposure to LPS

**Figure 4:**
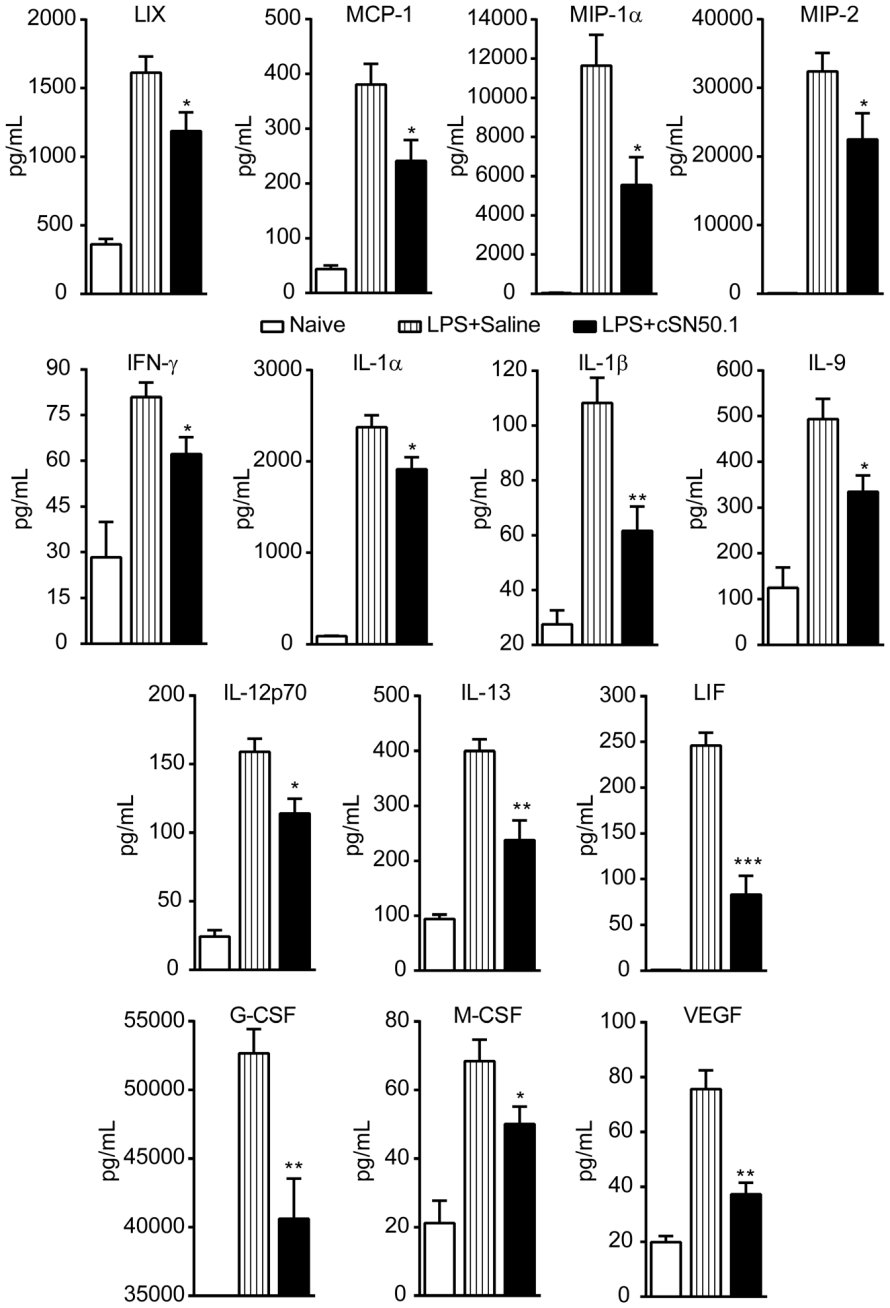
LPS-induced expression of chemokines, cytokines, and growth factors in the lung is suppressed by NTM. Fourteen cytokines, chemokines and growth factors elevated in BAL after direct airway exposure to LPS are significantly suppressed by NTM (cSN50.1 peptide) treatment. Data are presented as mean ± standard error, *n* = 4 naïve and 8–9 NTM- or saline-treated animals/group from 3 independent experiments. *p<0.05, **p<0.005, and ***p<0.0005 by Mann-Whitney *U* test comparing LPS-challenged groups.

We next sought to correlate our transcriptome analysis of primary macrophages with the production of inflammatory mediators in the bronchoalveolar space in a murine model of LPS-induced ALI. After challenging mice intranasally with LPS, we analyzed inflammatory mediators and cell populations in BAL fluid. We determined that i.p. administration of NTM suppressed the LPS-induced increase of 14 out of 32 proinflammatory chemokines, cytokines and growth factors analyzed in BAL fluid (Figure 4). Consistent with genomic reprogramming in primary macrophages (see Figure 1), NTM treatment effectively reduced chemokines linked to lung inflammation by their roles in mediating inflammatory cell migration to the lung: MCP-1/CCL2, MIP-1α/CCL3, and LIX/CXCL5. Suppressed production of cytokines IL-1α, IL-1β, IFN-γ, and IL-13 also correlated with results from BMDMs. Conversely, some inflammatory mediators, such as MIG, were not induced by direct airway exposure to LPS, while TNF-α, MIP-1β/CCL4, RANTES/CCL5 and IP-10/CXCL10 were induced by LPS but not suppressed by NTM treatment. However, these discrepancies between the qRT-PCR assay and BAL analysis can be attributed to the different cell populations present in each assay. NTM did reduce BAL levels of a number of other inflammatory mediators that were not analyzed by qRT-PCR: cytokines LIF, IL-9, and IL-12p70; chemokine MIP-2/CXCL2; and growth factors G-CSF, M-CSF, and VEGF, which is implicated in vascular permeability [28]. Overall, analysis of BAL documents the effectiveness of NTM in suppressing mediators of ALI after direct airway exposure to LPS.

**Figure 5:**
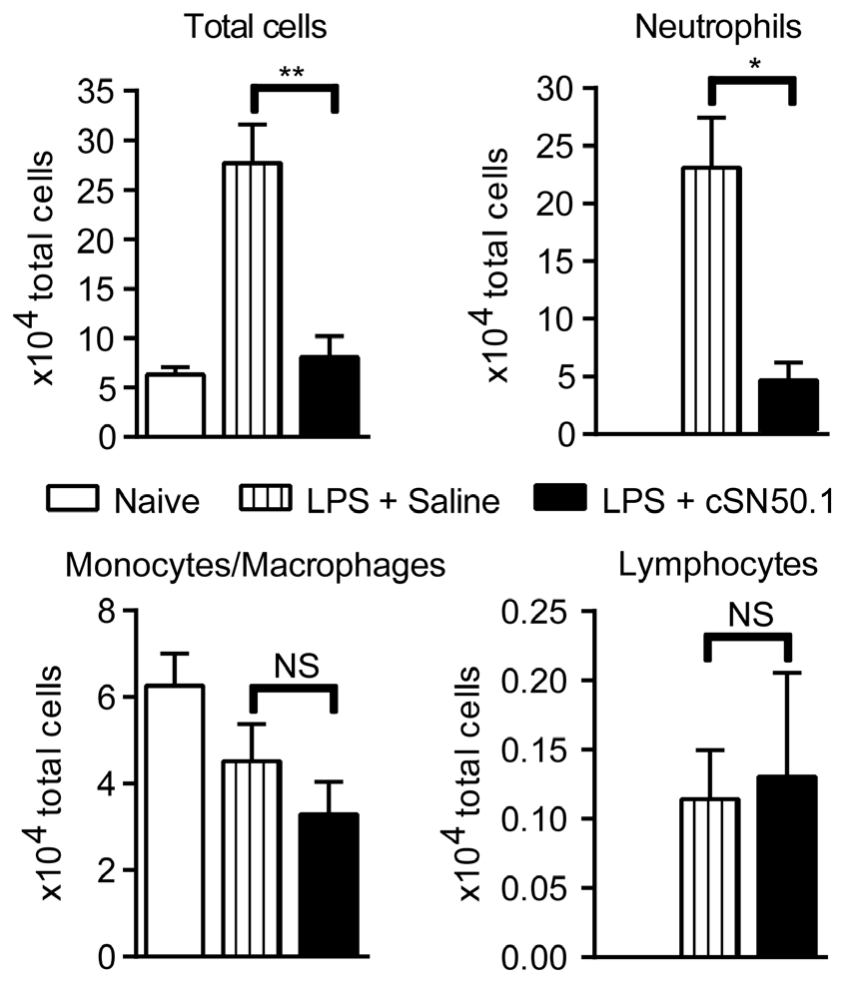
LPS-induced cellular trafficking to lungs is reduced in NTM-treated mice. Cell counts in BAL fluid collected from unchallenged mice (naïve) and 6 h after direct airway exposure to LPS, with i.p. NTM peptide (cSN50.1) or diluent control (saline) treatment. The LPS-induced increase in total cells is comprised primarily of neutrophils. Neutrophil trafficking to BAL is significantly reduced by NTM treatment while monocytes/macrophages and lymphocytes are not affected. Data are presented as mean ± standard error, *n* = 4 naïve and 5–7 NTM- or saline-treated animals/group from two independent experiments. *p<0.05, **p<0.005 by Mann-Whitney *U* test comparing LPS-challenged groups.

Suppression of chemokines MCP-1/CCL2, MIP-1α/CCL3, MIP-1β/CCL4, and LIX/CXCL5 in the inflammatory transcriptome analysis of NTM-treated primary macrophages and in BAL following direct airway exposure to LPS, led us to postulate that NTM would reduce leukocyte trafficking to the lung. As shown in Figure 5, we observed a 5-fold reduction in total cell count in BAL fluid from mice treated with NTM in comparison to the saline-treated control group (*p*<0.005), reducing the total cell count in BAL from NTM-treated mice to that of naive mice. A reduction in neutrophils (∼80%, *p*<0.05) accounted for most of the difference in BAL cellularity. Trafficking of monocytes/macrophages, the primary cell type detected in BAL fluid from naïve mice was moderately reduced (30%), but not significantly. Lymphocytes represented less than 1% of cells detected in BAL and their numbers were unchanged by NTM treatment (Figure 5). Thus, migration of neutrophils to the bronchoalveolar space in response to direct airway exposure to LPS, which evokes a robust expression of chemokine genes, is profoundly reduced by systemic administration of NTM. As neutrophils are the main source of oxidative stress to the delicate structure of the blood-air barrier [29], NTM’s suppression of chemokines responsible for massive neutrophil trafficking to the bronchoalveolar space may engender a new approach to lung cytoprotection. Thus, LPS-induced respiratory and systemic blood inflammatory responses and their lethal outcomes are averted.

## Discussion

Cumulatively, our results document the essential role of nuclear transport in development of a “genomic storm” and its sequelae induced by bacterial endotoxin. This evidence rests on the design and application of a highly soluble NTM, cSN50.1 peptide, which targets nuclear transport shuttles required for translocation of proinflammatory SRTFs and metabolism-regulating SREBPs. The emerging concept of a “genomic storm” in critically injured patients has been extended to human subjects challenged with bacterial endotoxin [10]. Our analysis of 46 genes encoding a wide array of mediators of inflammation (see Table 2) indicated that these genes are regulated by a group of transcription factors responsible for proinflammatory and metabolic signaling to the nucleus. As nuclear translocation of these transcription factors depends on nuclear transport machinery, we showed how modulation of this machinery with a cell-penetrating peptide targeting Imp α5 and Imp β1 calms the “genomic storm” triggered by bacterial endotoxin in primary BMDMs, and attenuates systemic and localized inflammation. These results obtained in murine macrophages are congruent with the gene expression profile of human peripheral blood leukocytes following endotoxin challenge [30]. Though it was recently asserted that humans and mice display discordant genomic responses to severe injury [31], subsequent reanalysis of the datasets employed in that study indicates that the gene expression profiles in the mouse models were highly similar to human responses [30].

We show that cSN50.1 had a profound suppressing effect on the inflammatory transcriptome of LPS-stimulated primary macrophages. This effect was paralleled by suppression of multiple cytokines, chemokines and, growth factors in plasma in response to LPS. Furthermore, NTM protected mice from lethality in two endotoxic shock models. NTM also attenuated proinflammatory cytokines and chemokines in inflamed lungs accompanied by reduced trafficking of neutrophils to the LPS-challenged bronchoalveolar space. These results indicate that the more soluble, next generation NTM modulates nuclear signaling induced by LPS, thereby effectively reducing its deleterious effects in systemic (endotoxic shock) and localized (lung) inflammation.

NTM is rapidly delivered (within 30–60 min after i.p. injection) through a receptor-and endocytosis-independent mechanism [20] to mouse blood cells and organs where it reaches sufficient intracellular concentration to engender localized (lung) and systemic (endotoxic shock) anti-inflammatory effects. Thus, the microvascular compartment of multiple organs that are vulnerable to LPS toxicity (*e.g.* lungs, liver, and kidneys) can be protected [18], [22]. Unlike anti-inflammatory glucocorticosteroids which produced discordant results in sepsis clinical trials [32], [33] and have potentially adverse side effects on metabolic balance (e.g. hyperglycemia and hyperlipidemia) [34]–[36], NTM not only suppresses pulmonary and systemic inflammation induced by LPS but also corrected metabolic derangements in an experimental model of hyperlipidemia by targeting nuclear transport of SREBPs [6]. This new function of cSN50.1 resulted in significant amelioration of hyperlipidemia and hyperglycemia. Metabolic dysregulation is a consequence of the systemic action of LPS, which in turn accelerates the vascular complications of hyperlipidemia [37]–[39]. SREBPs lack an NLS for binding to importins α and are shuttled to the nucleus by binding to Imp β1 [40]. We discovered that the SSHR motif of cSN50.1 binds Imp β1, thereby reducing nuclear translocation of SREBP-1 and −2. To the best of our knowledge, the immunoregulatory function of SREBP1 toward genes that encode proinflammatory cytokines and chemokines is not well understood.

Three interwoven mechanisms of LPS-induced systemic inflammation: endothelial injury, apoptosis, and microvascular dysfunction, depend on modulation of a nuclear transport-regulated genomic response [5], [22], [41], [42]. Significantly, we documented that systemic production of proinflammatory cytokines, chemokines and growth factors, which contribute to the subsequent development of endotoxic shock, was attenuated, while only IL-10 was increased by NTM, and only in plasma. Whereas elevation of IL-10 is notable in the profile of cytokine and chemokine responses in human sepsis studies [43]–[45], other critical proinflammatory mediators are consistently suppressed by NTM, and NTM treatment is highly protective against lethal shock. Since many small transcription factors (<45 kDa) essential to cell viability can traverse the nuclear pore without assistance from importins α and β [46], which are the targets of NTM [6], it is unlikely that NTM is involved in their nucleocytoplasmic trafficking. Thus, targeted modulation of nuclear transport of proinflammatory SRTFs, the master regulators of innate immunity and inflammation, and SREBPs, the master regulators of metabolic inflammation, offers a new approach to suppression of inflammatory, metabolic, and apoptotic mediators in the lung, liver, and other organs to interrupt rapidly progressing microvascular injury induced by bacterial endotoxin. Importantly, in polymicrobial sepsis and a pulmonary anthrax model, addition of NTM to antimicrobial therapy improved the outcome in terms of clearance of bacteria and survival [24], [26]. These studies militate against the notion that using NTM to modulate nuclear transport of SRTFs would compromise the outcome of microbial inflammatory diseases.

Important to these considerations of novel countermeasures toward LPS toxicity is the rise of multidrug-resistant Gram-negative bacterial infections in intensive care units throughout the United States, Europe, and Asia. These infections cause localized and systemic inflammation leading to septic shock and are a growing concern in immunocompromised hosts [47], [48]. Alarmingly, Gram-negative bacteria are isolated from 62% of patients with severe sepsis; approximately half of these cases result in individuals dependent upon mechanical ventilation [49]. The rate of acquiring bacterial pneumonia increases up to 21% in intubated patients and rises even higher as the length of intubation persists [50]–[52]. In total, Gram-negative bacteria account for more than 30% of hospital-acquired infections causing pneumonia [53].

A cardinal feature of localized inflammation of the lungs (pneumonia and ARDS) caused by Gram-negative bacteria is endothelial and epithelial injury [29]. The response to microbial virulence factors leads to uncontrolled production of proinflammatory chemokines and cytokines that contribute to collateral damage of the air-blood barrier. We postulate that simultaneous reduction of multiple proinflammatory cytokines and chemokines induced by direct airway exposure to LPS has a salutary effect on other lung-associated inflammatory cells. For example, intracellular delivery of NTM significantly reduced trafficking of neutrophils to the bronchoalveolar space. At least three NTM-suppressed cytokines, IL-6, IL-17, and IFN-γ, through their localized action, contribute to the disruption of lung endothelial and epithelial barriers [54], [55], manifested by migration of leukocytes to the bronchoalveolar space, leakiness of plasma proteins therein, and impairment of respiratory function. Consistent with localized suppression of LIX, MCP-1, MIP-1α, and MIP-2 by NTM, a significant migration of neutrophils to the bronchoalveolar space was attenuated, thereby reducing potential oxidant injury to the respiratory epithelium and vascular endothelium. Systemic suppression by NTM of chemokines IP-10, MCP-1, MIG, MIP-1β and RANTES in plasma is also of significance due to their role in induction of inflammatory cell migration to the bronchoalveolar space [56], [57].

The need for new therapeutic approaches to protect lungs and other major organs from LPS-induced injury is apparent as there is currently no available FDA-approved drug to counteract collateral organ injury during sepsis, ALI, and ARDS [11]. The prospect for an effective countermeasure based on a single cytokine/chemokine target for monoclonal antibodies or soluble cytokine receptor antagonists [58]–[60] is dimmed by the potential for significant redundancy in cytokine signaling. We have now demonstrated the beneficial effects of NTM in two diverse models of localized lung inflammation: one induced by LPS, a potent agonist of TLR4-expressing myeloid, endothelial, and epithelial cells as documented in this study, and the second, induced by staphylococcal enterotoxin B, a superantigenic immunotoxin, which is a robust agonist of T cell receptor-expressing cells [18]. Our findings document the potential utility of targeting the nuclear transport shuttles with cell-penetrating peptides to simultaneously suppress production of multiple cytokines and chemokines and potentially correct metabolic derangements [6]. Survivors of sepsis have strikingly lower levels of NF-κB in the nuclear compartment of peripheral blood mononuclear leukocytes than non-survivors [61]. Using an established formula for extrapolating a human equivalent dose from the animal dose through normalization to body surface area [62], the effective cSN50.1 peptide dose of 0.66 mg/20 g mouse translates to a manageable human dose of 200 mg/70 kg. This is similar to a standard oral dose of ibuprofen, a non-steroidal anti-inflammatory drug, which at a daily intravenous dose of 800 mg, was proven ineffective in reducing shock, ARDS, and mortality in a human sepsis trial [63]. However, further studies will be required to determine the pharmacokinetics, toxicity, and therapeutic efficacy of NTMs.

Given its efficient delivery to the lungs and rapid action, a cell-penetrating NTM peptide targeting nuclear import of SRTFs and SREBPs may represent a much-needed adjunctive therapy to complement antimicrobials that target Gram-negative bacteria in systemic (endotoxic shock) and localized (lung) infections. In such a combined treatment, antimicrobials would limit the proliferation of Gram-negative bacteria that shed LPS and express exotoxins while the collateral damage to lungs and other organs through uncontrolled inflammation would be contained by NTM. Thus, the serious and costly consequences of Gram-negative infections can be potentially reduced.
